# CqsA/LuxS-HapR Quorum sensing circuit modulates type VI secretion system VﬂT6SS2 in *Vibrio fluvialis*

**DOI:** 10.1080/22221751.2021.1902244

**Published:** 2021-03-30

**Authors:** Xiaoshu Liu, Jingjing Pan, He Gao, Yu Han, Anran Zhang, Yuanming Huang, Ping Liu, Biao Kan, Weili Liang

**Affiliations:** State Key Laboratory for Infectious Disease Prevention and Control, Chinese Center for Disease Control and Prevention, National Institute for Communicable Disease Control and Prevention, Beijing, People’s Republic of China

**Keywords:** Type VI secretion system (T6SS), quorum sensing (QS), LuxO, HapR, *Vibrio fluvialis*

## Abstract

*Vibrio fluvialis* is an emerging enteric pathogen of increasing public health threat. Two quorum sensing (QS) systems, VfqI-VfqR and CqsA/LuxS-HapR, and two type VI secretion systems (T6SSs), VflT6SS1 and VflT6SS2, have been identified in *V. fluvialis*. Whether there exists any correlation between the two systems is unclear. In this study, we found that CqsA/LuxS-HapR circuit regulator LuxO represses while HapR activates VﬂT6SS2. The effect of LuxO is more pronounced at low cell density and is HapR-dependent. Deletion of *hapR* abolished Hcp expression and alleviated antibacterial virulence. However, these effects were rescued by HapR-expressing plasmid. Reporter fusion analyses showed that HapR is required for the promoter activities of VﬂT6SS2. Sequence inspection of the major cluster promoter revealed two potential Motif 1 HapR binding sites, and their bindings to HapR were confirmed by both electrophoretic mobility shift assay (EMSA) and DNase I footprinting assay. Meanwhile, two single Motif 2 sites were identified in *tssD*2_a (*hcp*A) and *tssD*2_b (*hcp*B) promoter regions of the orphan cluster which are less conserved and displayed lower affinities to HapR. Together, our study demonstrated that CqsA/LuxS-HapR QS manipulate VflT6SS2 in *V. fluvialis*, and this finding will enhance our understanding of possible crosstalk between T6SS and QS in microbes.

## Introduction

*Vibrio fluvialis* is a halophilic, polarly-flagellated, Gram-negative bacterium globally distributed and commonly found in marine, estuarine environments, and seafood. It was first isolated from a patient with severe diarrhoea in 1975 in Bahrain [1] and once called Group F vibrios and EF-6 vibrios [2]. *V. fluvialis* is considered as an emerging foodborne pathogen and is common in infants, children, and young adults [3]. *V. fluvialis* associated sporadic cases and outbreaks of acute gastroenteritis have been reported from different parts of the world [4–6]. *V. fluvialis* also causes extra-intestinal infections in human and is pathogenic to cultured fish and lobsters, resulting in serious economic losses [5–8]. The clinical symptoms of *V. fluvialis* gastroenteritis are very similar to that of *V. cholerae* except for the frequent presence of erythrocytes/blood in stool of *V. fluvialis* infection [9]. Furthermore, *V. fluvialis* is prone to acquire antibiotic resistance genes through mobile genetic elements even without any antibiotic selective pressure [10,11]. The increasing incidence of multidrug resistant *V. fluvialis* mediated by SXT element, plasmids and integrons has been reported and is becoming a major public health challenge [4,11–15].

The type VI secretion system (T6SS) is a newly discovered secretion system in Gram-negative bacteria [16,17]. It is a contractile weapon that injects toxic effector proteins into target cells through contact-dependent manner and thus kills neighbouring prokaryotic and eukaryotic organisms [18–21]. T6SS plays a critical role in bacterial virulence and competitive environmental survival among bacteria in the same niche [18–21] and undergoes a series of complicated regulation which could occur at transcriptional, post transcriptional, translational, and posttranslational levels [22]. In previous work, we identified two T6SS encoding gene clusters, namely VflT6SS1 and VflT6SS2, in a clinical *V. fluvialis* isolate 85003[23]. VflT6SS1 is inactive under laboratory conditions, while VflT6SS2 is active and mediates inter-bacterial antagonistic interactions [23]. VflT6SS2 secretion activity is growth-phase dependent and optimally induced at low and warm temperatures, as well as high osmolarity conditions [23]. However, key factors that mediate these environmental signals remain to be identified. Integration host factor (IHF) and σ^54^-dependent VasH encoded in VflT6SS2 major cluster transcriptionally activate the expression of VflT6SS2. IHF targets both the major and orphan clusters whereas VasH acts on the orphan cluster [23,24]. In addition to IHF and VasH, many other global transcriptional regulators such as sigma factor 54 (σ^54^, RpoN), cAMP receptor protein (CRP), and TsrA have also been reported to manipulate T6SS expression and function [25–27].

Quorum sensing system (QS) is a density-dependent communication system employed by host bacteria to sense and to respond to single or a variety of signal molecules secreted by itself and surrounding bacteria to synchronize target gene expressions, thus participating in the regulation of its own various physiological functions, including the production of extracellular enzymes, pigments and toxins, expression of virulence genes, biofilm formation, bioluminescence, antibiotic resistance, bacterial dynamics, and so on [28–33]. QS system was first found in marine *Vibrio fisheri* [33–35], and later discovered in a variety of bacteria including Gram-positive and Gram-negative ones [28–32].

Up to now, two QS systems have been identified in *V. fluvialis*: one is VfqI-VfqR system with acyl homoserine lactone (AHL) as the self-inducible signal molecule, and the other is CqsA/LuxS-HapR system with cholerae autoinducer 1 (CAI-1) and autoinducer 2 (AI-2) as self-inducing signal molecules [36]. VfqI/VfqR is a LuxI/LuxR homologue which is considered as the QS model in Gram-negative bacteria. VfqI primarily produces 3-oxo-C10-HSL which is sensed by VfqR and subsequently activates *vfqI* expression [36]. Similar to those in *V. cholerae* and *V. harveyi*, the signals of CAI-1 and AI-2 molecules in *V. fluvialis* are ultimately transmitted to the QS master regulator HapR via a shared LuxU/LuxO involved pathway. CAI-1 or AI-2 alone is sufﬁcient to activate *hapR* expression [36]. The QS of *V. fluvialis* plays an important role in pathogenesis by regulating several potential virulence factors, such as an extracellular protease, haemolysin and others [36].

In this study, we investigated functional association between QS and T6SS in *V. fluvialis* by focusing on the regulatory role of CqsA/LuxS-HapR QS circuit on VflT6SS2 function. We firstly demonstrated that LuxO and HapR, two members of CqsA/LuxS-HapR pathway, respectively represses and activates the expression and secretion of Hcp, a hallmark of active T6SS. Subsequently, we elucidated that the effect of LuxO on Hcp is HapR-dependent. Deletion of *hapR* completely abolished Hcp expression and secretion, and promoted *Escherichia coli* survival in competition assay. However, these Δ*hapR*-associated effects could be restored by introducing HapR-expressing plasmid. Further studies revealed that HapR physically binds to the promoter regions of both major and orphan clusters of VﬂT6SS2, leading to the transactivation of VflT6SS2. In all, our current study disclosed that QS circuit plays a crucial role in the regulation of T6SS function in *V. fluvialis*.

## Materials and methods

### Bacterial strains, culture conditions, and plasmids

All bacterial strains and plasmids used in this study are listed in Table 1. The wild-type (WT) of *V. fluvialis* 85003 and its derivative mutants were usually grown in Luria–Bertani (LB) broth (pH7.4) with 1% NaCl (170 mM) at 30°C. *E. coli* strain DH5α*λpir* and SM10*λpir* were cultured at 37°C for cloning and mating purposes. When required, culture media were supplemented with antibiotics at the following final concentrations: streptomycin (Sm, 100 μg/mL), ampicillin (Amp, 100 μg/mL), tetracycline (Tc, 10 μg/mL for *E. coli*, 2.5 μg/mL for *V. fluvialis*), chloramphenicol (Cm, 10 μg/mL), rifampicin (Rfp, 50 μg/mL), kanamycin (Km, 50 μg/mL), or with isopropyl-b-D-thiogalactopyranoside (IPTG) at a final concentration of 0.5 mM.

**Table 1.** Bacteria strains and plasmids used in this study.

Strain/Plasmid	Characteristics	Reference/Source
*E. coli*		
SM10*λpir*	*thr thi tonA leu supE lacY recA*::RP4-2Tc::Mu (*λpir*R6 K), Km^R^	Mekalanos Laboratory (Harvard Medical School)
DH5α*λpir*	F-D(*lacZYA-argF*)U169*recA endA1 supE44 relA1λ::pir*	Laboratory stock
MG1655	K-12 F^–^ λ^–^*ilvG^–^ rfb-50 rph-1*, Rfp^R^	Laboratory stock
C43(DE3)	F^–^*ompT hsdSB* (rB^-^mB^-^) *gal dcm* (DE3)	Laboratory stock
*V. ﬂuvialis* 85003		
WT	*V. fluvialis*, wild type, Sm^R^	Laboratory stock
Δ*hapR*	85003 with *hapR* in-frame deletion, Sm^R^	[36]
Δ*cqsA*	85003 with *cqsA* in-frame deletion, Sm^R^	This study
Δ*luxS*	85003 with *luxS* in-frame deletion, Sm^R^	[36]
Δ*luxU*	85003 with *luxU* in-frame deletion, Sm^R^	[36]
Δ*luxO*	85003 with *luxO* in-frame deletion, Sm^R^	[36]
Δ*luxOhapR*	85003 Δ*luxO* with *hapR* in-frame deletion, Sm^R^	This study
Plasmid		
pBBR*lux*	promoterless of *luxCDABE*, Cm^R^	Laboratory stock
pWM91	Suicide vector containing R6 K *ori*, *sacB*, *lacZα*; Amp^R^	Laboratory stock
pSRKTc	Broad-host-range vector containing *lac* promoter, *lacI*^q^, *lacZ*α,Tet^R^	Laboratory stock
pET30a	expression vector containing f1 *ori*, *lacI*; Kan^R^	Laboratory stock
pVflT6SS2-*lux*	Promoter region of *tssB*2 in pBBR*lux*	[24]
p*tssD*2a-*lux*	Promoter region of *tssD*2_a in pBBR*lux*	[24]
p*tssD*2b-*lux*	Promoter region of *tssD*2_b in pBBR*lux*	[24]
p*tssD*2c-*lux*	Promoter region of *tssD*2_c in pBBR*lux*	[24]
pSR*hapR*	618 bp *hapR* ORF of *V. fluvialis* in pSRKTc	This study
pSR*vasH*	1.596 kb *vasH* ORF of *V. fluvialis* in pSRKTc	[23]
pET*hapR*	615 bp *hapR* ORF of *V. fluvialis* in pET30a	This study
pWMΔ*cqsA*	1.319 kb *Xho*I-*BamH*I Δ*cqsA* fragment of *V. ﬂuvialis* in pWM91	This study
pWMΔ*hapR*	1.017kp *Xho*I-*Not*I Δ*hapR* fragment of *V. ﬂuvialis* in pWM91	[36]

### Construction of mutants and complementation plasmids

*V. fluvialis* Δ*hapR*, Δ*luxS*, Δ*luxU* and Δ*luxO* mutants were constructed previously by allelic exchange using 85003 as a precursor [36]. Δ*cqsA* and Δ*luxO*Δ*hapR* were constructed in this study. Briefly, chromosomal DNA sequences flanking *cqsA* open reading frame (ORF) were amplified by polymerase chain reaction (PCR) using high-fidelity polymerase (Prime STAR, TaKaRa, Dalian, China) and stitched together by overlapping PCR as described previously [37]. The resultant fragment was cloned into pWM91 suicide plasmid to generate pWMΔ*cqsA*. The recombinant plasmids, pWMΔ*cqsA* and pWMΔ*hapR*, were respectively mobilized into *V. fluvialis* WT and Δ*luxO* strains from *E. coli* SM10*λpir* by conjugation. Amp and Sm resistant exconjugants were counter-selected by streaking on NaCl-free LB agar containing 10% sucrose. Sucrose-resistant colonies were tested for Amp sensitivity, and mutant allele was verified by PCR and further confirmed by sequencing. HapR complementation plasmid pSR*hapR* was constructed by cloning the 85003 WT *hapR* coding sequences into pSRKTc which was amplified with primers hapR-*Nde*I and hapR-*XhoI*-1 (Table S1). The HapR were expressed from the *lac* promoter with the induction of IPTG.

### RNA extraction and quantitative real-time PCR (qRT-PCR)

*V. fluvialis* strains were cultured to the desired cell density. Total RNA extraction and cDNA synthesis were performed as described previously [37]. PCR was performed by CFX96 (Bio-Rad) using SYBR Premix Ex Taq (TaKaRa, Dalian, China). Relative expression values (*R*) were calculated using the equation $R=2^{-(\Delta C_{T}\;target-\Delta C_{T}\;reference)}$, in which C_T_ is the fractional threshold cycle. *recA* was used as reference and primers used for examining *recA* and *hcp* (*tssD*2) were listed in Table S1. A control reaction with extracted RNA instead of cDNA as a template was performed for each sample to exclude contamination from chromosomal DNA.

### Analyses of VflT6SS2 expression and secretion

Overnight cultures of *V. fluvialis* 85003 WT and its derivative mutants were inoculated at 1:100 into 20 mL LB and incubated to respective OD_600_ of 0.2, 0.5, 1.0 and 1.5 with shaking at 30°C. Protein samples from cell pellets and cell-free supernatants were prepared as previously described [23,24]. The protein concentration was determined using the BCA^TM^ protein assay kit (Thermo Fisher Scientiﬁc, United States). Western blot analysis was performed with polyclonal rabbit anti-Hcp antibody and anti-*E. coli* cyclic AMP receptor protein (CRP) antibody (BioLegend, United States) [23].

For *hapR* complementation, overnight cultures of Δ*hapR* mutant containing plasmid pSR*hapR* (Δ*hapR*/pSR*hapR*) or pSRKTc (Δ*hapR*/pSRKTc) were grown in LB with tetracycline to OD_600_ of 0.5. Each culture was then divided in half. One half was induced with IPTG (ﬁnal concentration of 0.5 mM), and the other half was used as a control. The cultures were continually incubated to OD_600_ 1.5 at 30°C with shaking.

### Bacterial killing assay

Bacterial killing assay was performed as described previously [23,24] and used to evaluate the antibacterial virulence of *V. fluvialis*. The predator *V. fluvialis* strains WT/pSRKTc (WT with plasmid pSRKTc), Δ*hapR*/pSRKTc (Δ*hapR* with plasmid pSRKTc) and Δ*hapR*/ pSR*hapR* (Δ*hapR* with complementation plasmid pSR*hapR*) were mixed with *E. coli* prey MG1655 at a 9:1 ratio (predator: prey) in triplicates. A total of 10 µL of the mixtures were spotted on filter membrane on LB agar plates with 2% NaCl and 0.5 mM IPTG, and incubated at 30°C for 5 h. The colony-forming units (CFU) of the prey *E. coli* MG1655 at the beginning (T0) and after 5 h incubation with predator (T5) were calculated by plating 10-fold serial dilutions on Rfp resistant agar plates. The CFU of *V. fluvialis* predators were determined on Tc resistant agar plates.

### Luminescence activity assay

The overnight culture of *V. fluvialis* strain containing *lux* reporter fusion plasmid was diluted at 1:100 in fresh LB and was incubated at 30°C with shaking. Luminescence was measured every one hour by transferring 200 µL aliquots of each culture into an opaque-wall 96-well microtiter plate (Thermo Fisher Scientiﬁc, United States). The OD_600_ and luminescence were measured with a microplate reader (Infinite M200 Pro, Tecan, Austria). Luminescence activity is calculated as light units/OD_600_ as previously described [24].

### Expression and purification of HapR

*hapR* ORF was amplified by PCR with primers hapR-*Nde*I and hapR-*Xho*I (Table S1) and cloned into pET30a at *Nde*I*/Xho*I sites to generate pET*hapR*. *E. coli* strain C43 (DE3) containing the resultant expression plasmid pET*hapR* was cultured to OD_600_ of 0.5-0.6 with shaking at 30°C and induced with 1 mM IPTG for 3 h. The 6× His-tagged HapR protein was purified using affinity chromatography Ni^+^ resin (Thermo Fisher Scientiﬁc, United States) according to the manufacturer’s instructions. The elution sample was dialyzed overnight at 4°C against 1× PBST and then centrifuged at 12,000 rpm for 15 min at 4°C in centrifugal filter to concentrate the proteins.

### Electrophoretic mobility shift assay (EMSA)

The 375 bp probes for *tssD*2_a (*hcpA*) and *tssD*2_b (*hcpB*) promoter regions were amplified with primer pairs HcpA-up-Biotin/HcpA-dn-Biotin and HcpB-up-Biotin/HcpA-dn-Biotin with plasmids p*tssD*2a-*lux* and p*tssD*2b-*lux* as templates, respectively. The 450 bp probe for VflT6SS2 major cluster promoter was amplified with primer pair T6SS2-up-Biotin/T6SS2-dn-Biotin using plasmid pVﬂT6SS2-*lux* as a template [24]. The reaction mixture of 20 ng biotin-labelled probe with increasing amounts of purified HapR protein in reaction buffer (10 mM Hepes, 100 mM KCl, 0.2 mM EDTA, 2 mM DTT, 10% glycerol, pH 7.9) together with 100 ng BSA and 100 ng dI-dC in each reaction (20 μL) was incubated at room temperature for 30 min, and then separated on a 6% native polyacrylamide gel. The free and HapR-bound probes were visualized with the Chemiluminescent Nucleic Acid Detection Module (Thermo Fisher Scientiﬁc, United States) according to the manufacturer's instruction after transferring onto nylon membranes.

### DNase I footprinting assay

The procedures of DNase I footprinting and sequencing assays were carried out as previously described [38]. Briefly, the promoter regions of *tssB*2 (*vipA*) and *tssD*2_a (*hcpA*) were amplified by PCR using the primer pairs of vipA-M13F1-FAM/vipA-M13R-HEX and hcpA-M13F1-FAM/ hcpA-M13R-HEX (Table S1), respectively. The resultant products (i.e. FAM- or HEX-labelled DNA probes) were incubated with the increasing amounts of HapR protein in the same binding buffer as in EMSA, and then digested by the optimized RQ1 RNase-Free DNase I (Promega, United States). The digested DNA fragments were analysed by ABI 3500XL DNA Genetic analyser with GeneMarker software 2.2, while the DNA sequencing products were surveyed with Sequence Scanner software v1.0.

## Results

### CqsA/LuxS-HapR QS circuit regulates the expression and function of VflT6SS2

**Figure 1.**
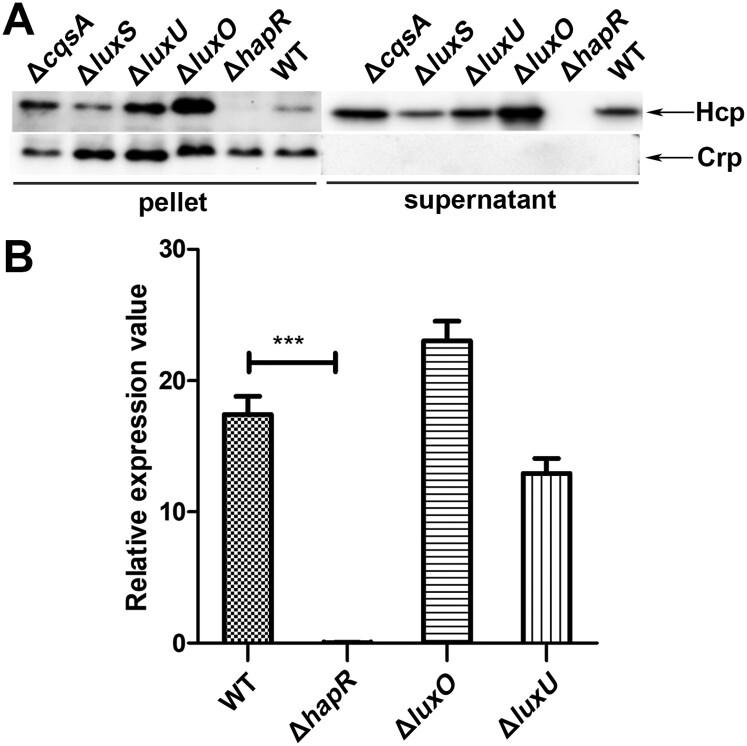
CqsA/LuxS-HapR QS circuit modulates VflT6SS2 function. (A) Expression and secretion of Hcp in *V. ﬂuvialis* 85003 wild type (WT), Δ*cqsA*, Δ*luxS*, Δ*luxU*, Δ*luxO*, and Δ*hapR* mutants. Strains were cultured at 30°C in LB medium to OD_600_ 1.5. Western blot analysis was performed with 7 μg of total protein extract from cell pellets or culture supernatants to detect the target protein levels using the anti-Hcp and anti-Crp antibodies. The arrows show the immunoblot bands to Hcp or Crp. The absence of Crp protein in the supernatant indicates that the supernatant Hcp was not from cell lysis. (B) Comparison of the mRNA level of *hcp* (*tssD*2) in *V. ﬂuvialis* 85003 WT, Δ*hapR*, Δ*luxO*, and Δ*luxU* mutants. *V. fluvialis* strains were grown at 30°C in LB medium to OD_600_ 1.5. Total RNA was extracted, and the mRNA abundance of *hcp* (*tssD*2) was determined by qRT-PCR. The data represent three biological repetitions. Statistical significance was determined by unpaired two-tailed Student’s *t* test compared to the WT. *** *P* = 0.0002.

To determine whether CqsA/LuxS-HapR QS affects the function of VflT6SS2 in *V. fluvialis*, we examined the expression and secretion of Hcp in CqsA/LuxS-HapR circuit gene deletion mutants, including Δ*cqsA*, Δ*luxS*, Δ*luxU*, Δ*luxO*, and Δ*hapR*. As shown in Figure 1(A), no Hcp was detected in both cell pellet and culture supernatant of Δ*hapR* mutant, indicating *hapR* is necessary to maintain the expression and function of VflT6SS2. The expressions of Hcp in cell pellets of Δ*luxU* and Δ*luxO* mutants were obviously upregulated compared to that in WT, suggesting repressive effects of both *luxU* and *luxO* products. Though similar elevated trends were observed for Hcp in their culture supernatants, the increase was more apparent in Δ*luxO* than in Δ*luxU*. We noticed that deletion of *cqsA* or *luxS* alone did not greatly affect the expression and secretion of Hcp, implying the possible redundancy of CAI-1 and AI-2 signals in *V. fluvialis*. Of note, this result is consistent with our earlier findings which showed that the *hapR* expression remains high in Δ*cqsA* or Δ*luxS* mutant but drastically decreased in Δ*cqsA*Δ*luxS* double mutant [36]. These results primarily showed that CqsA/LuxS-HapR QS pathway participates in the regulation of VflT6SS2 function.

To further confirm the above outcomes, the same cultures of Δ*luxU*, Δ*luxO*, Δ*hapR*, and WT used for Western blotting were also applied to detect the mRNA level of *hcp* (*tssD*2). As shown in Figure 1(B), the mRNA level of *hcp* (*tssD*2) is extremely lower in Δ*hapR* than in WT. However, in Δ*luxO* or Δ*luxU* mutant, although the *hcp* (*tssD*2) mRNA levels were a little higher or lower comparing to their WT respectively, no statistical difference was reached for both of them. Collectively, these results showed that CqsA/LuxS-HapR QS signaling pathway was involved in the regulation of VflT6SS2, especially, both *luxO* and *hapR* play important roles during this process.

### LuxO regulates VflT6SS2 function at low cell density

**Figure 2.**
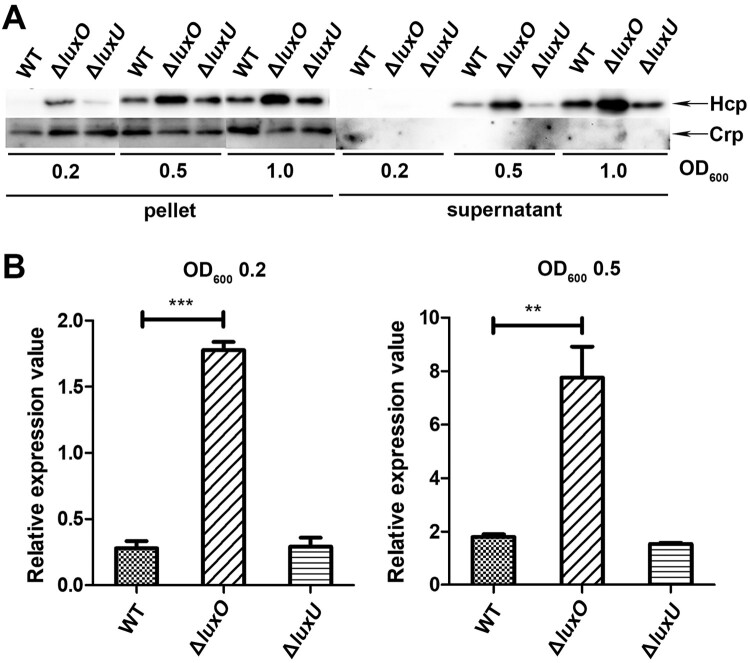
LuxO inhibits VflT6SS2 function at low cell density. (A) Hcp expression and secretion in *V. ﬂuvialis* 85003 WT, Δ*luxU* and Δ*luxO* mutants under different cell growth densities. Strains were incubated at 30°C in LB medium to OD_600_ of 0.2, 0.5, or 1.0. Then, Western blot analysis was performed with 7 μg of total protein extract from cell pellets or supernatants. The arrows indicate the immunoblot bands of the Hcp and Crp proteins. (B) Determination of the mRNA level of *hcp* (*tssD*2) in *V. fluvialis* WT, Δ*luxO*, and Δ*luxU* mutants. The strains were grown to OD_600_ 0.2 or 0.5, and then the total RNA was extracted from the culture and the *hcp* (*tssD*2) mRNA abundance was determined by qRT-PCR. The data represent three biological repetitions. Statistical significance was determined by unpaired two-tailed Student’s *t* test. ***P = 0.0005; **P = 0.0066.

It is commonly accepted that, the response regulator LuxO is activated at low cell density through phosphorylation relay from LuxU and is inactivated at high cell density when auto-inducers accumulate to high levels which leads to a reversed phosphate flow [31,39]. Our experiments in Figure1 were performed at OD_600_ around 1.5, a high cell density, which may alleviate the deletion effect of *luxO* and *luxU* genes. To better dissect the regulation of QS on VflT6SS2, we further detected the expression and secretion of Hcp in WT, Δ*luxU*, or Δ*luxO* mutant under different cell densities, specifically, OD_600_ of 0.2, 0.5, and 1.0. As depicted in Figure 2(A), under these lower cell culture densities, the expression and secretion of Hcp in Δ*luxO* were clearly higher than those of in WT except no detectable secretion was observed at OD_600_ 0.2 in both strains probably because of low Hcp expression. However, the increase of Hcp expression and secretion in Δ*luxU* was not as dramatic as in Δ*luxO*, and the underlying mechanism deserves to be investigated. We also measured *hcp* (*tssD*2) mRNA level at OD_600_ 0.2 and 0.5 (Figure 2(B)) in WT, Δ*luxU*, and Δ*luxO*, and found that the alteration trends for *hcp* (*tssD*2) mRNAs were in accordance with their protein levels. Furthermore, we found that with the increment of cell culture densities, the increase amplitudes of *hcp* (*tssD*2) mRNA gradually decreased as marked by an induction fold changes from 6.5 (Δ*luxO/*WT: 1.81/0.27), 4.3 (Δ*luxO/*WT: 7.76/1.80), to 1.3 (Δ*luxO/*WT: 23.01/17.42) at OD_600_ of 0.2, 0.5, and 1.5, respectively. These results indicated that LuxO was involved in the negative regulation of VflT6SS2 function at low cell density.

## HapR is a critical regulator of CqsA/LuxS-HapR QS on VflT6SS2 function

**Figure 3.**
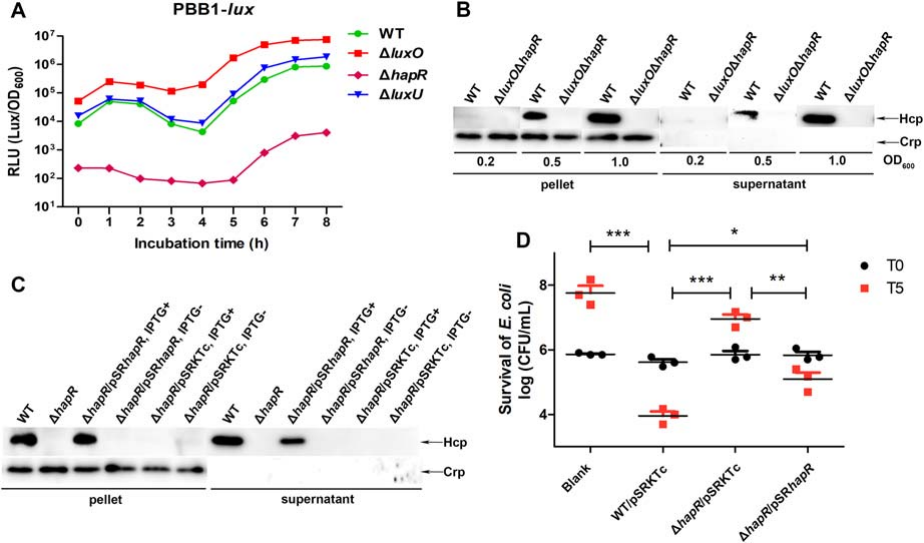
LuxO represses VflT6SS2 function in a HapR-dependent manner. (A) Luminescence activity of pBB1 in *V. ﬂuvialis* 85003 WT and designated mutants. The overnight cultures of *V. ﬂuvialis* strains containing pBB1 harbouring a *V. harveyi luxCDABE* operon were 1:100 diluted in LB medium and incubated at 30°C with shaking. The luminescence was measured by transferring 200 µL aliquots of each culture into an opaque-wall 96-well microtiter plate. Luminescence activity is calculated as light units/OD_600_. (B) Hcp expression and secretion in wild type and Δ*luxO*Δ*hapR* double mutant strains under different cell growth densities. Strains were incubated at 30°C in LB medium to OD_600_ of 0.2, 0.5, or 1.0. Western blotting was performed with 7 μg of total protein extract from cell pellets or supernatants. The bands for Hcp and Crp were marked with arrows. (C) HapR restoration on Hcp expression and secretion. Strains for WT, Δ*hapR*, or different combinations of Δ*hapR* and its complementary or empty vector were cultured with or without IPTG induction*.* Western blot analysis was performed with 7 μg of total protein extract from cell pellets or supernatants. The detailed strain culture condition and sample preparation were described in the “Materials and Methods.” The arrows show the immunoblot bands to Hcp or Crp. (D) Bacterial killing assay between *V. fluvialis* and *E. coli* strain MG1655. *V. fluvialis* strains containing designated plasmids were cultured at 30°C in the presence of IPTG and then used as predators and mixed with prey *E. coli* MG1655 at a 9:1 ratio (predator: prey) in triplicates. Bacterial killing assay was performed as described in the “Materials and Methods.” The CFU of the prey *E. coli* strain MG1655 was determined at the start point (T0) and after 5 h (T5) co-culture with *V. ﬂuvialis* predators. Statistical significance between sample groups at T5 was determined by unpaired two-tailed Student’s *t* test. Blank, medium only. ***P = 0.0001; **P = 0.0017; *P = 0.0104.

Previously, we showed that LuxO has a negative effect on *hapR* expression [36], thus we reasoned that the highly increased expression of Hcp in Δ*luxO* is mediated by HapR. To test our hypothesis, firstly we indirectly measured the HapR level in WT and several QS circuit member deletion mutants of *V. fluvialis* by introducing the pBB1 cosmid, which carries the *V. harveyi luxCDABE* operon and is activated specifically by LuxR (homolog of HapR) or HapR to induce luminescence. Consequently, the intensity of luminescence is positively correlated to the level of LuxR or HapR. Our results disclosed that the Δ*luxO* and Δ*hapR* mutants respectively possessed the highest and lowest light production curves comparing to WT and Δ*luxU*, which owned similar light curves (Figure 3(A)). Noticeably, the curves in Δ*luxU* and WT displayed similar characteristic V-shape, implying a trend of gradually reduced luminescence from diluted overnight culture to the steady rise during the accelerated bacterial growth thereafter. In a word, the highest luminescence curve in Δ*luxO* was an indicative of highest HapR level, denoting a likely reverse correlation between LuxO and HapR.

To further clarify the above results, we constructed a double deletion mutant Δ*luxO*Δ*hapR* and measured Hcp expression in this mutant under different cell culture densities, and compared it to its wild-type counterpart. As shown in Figure 3(B), no matter what the OD_600_ is, the Δ*luxO*Δ*hapR* strain completely lost the ability to express and secrete Hcp compared to its WT, supporting the notion that the expression and secretion of Hcp in Δ*luxO* completely rely on HapR.

To exclude the potential polarity effect of *hapR* deletion, we performed trans-complementation assays by introducing complementation plasmid pSR*hapR* into Δ*hapR* strain. As demonstrated in Figure 3(C), when induced by IPTG, the expression and secretion of Hcp were restored in Δ*hapR*/pSR*hapR* but failed if without IPTG induction. As to Δ*hapR*/pSRKTc strain, no Hcp expression and secretion were detected both in the presence and absence of IPTG induction. Collectively, these results supported that HapR is indeed a key regulator of CqsA/LuxS-HapR QS circuit on VflT6SS2 function in *V. fluvialis*.

Lastly, we performed bacterial killing assay by employing *E. coli* MG1655 as prey. As shown in Figure 3(D), the colony-forming ability of the prey was retained when co-cultured with Δ*hapR*/pSRKTc but reduced when with WT/pSRKTc or Δ*hapR/*pSR*hapR*. To further exclude the probable impact of nutrition competition due to fast growth of WT/pSRKTc or Δ*hapR*/pSR*hapR* on the decrease of co-cultured *E. coli*, we measured the growth curve of each strain in LB and further determined the CFU of *V. fluvialis* predators at the start point (T0) and after 5 h (T5) co-culture with prey MG1655. As shown in Figure S1, all *V. fluvialis* strains displayed similar growth trends (Figure S1(A)) and there was no much difference between the CFU of different *V. fluvialis* predators (Figure S1(B)) after 5 h incubation with strain MG1655. Together, these results validated that HapR contributes to the competitive fitness of *V. fluvialis*, most likely through activating VflT6SS2-mediated bactericidal activity.

### HapR transcriptionally activates VflT6SS2

**Figure 4.**
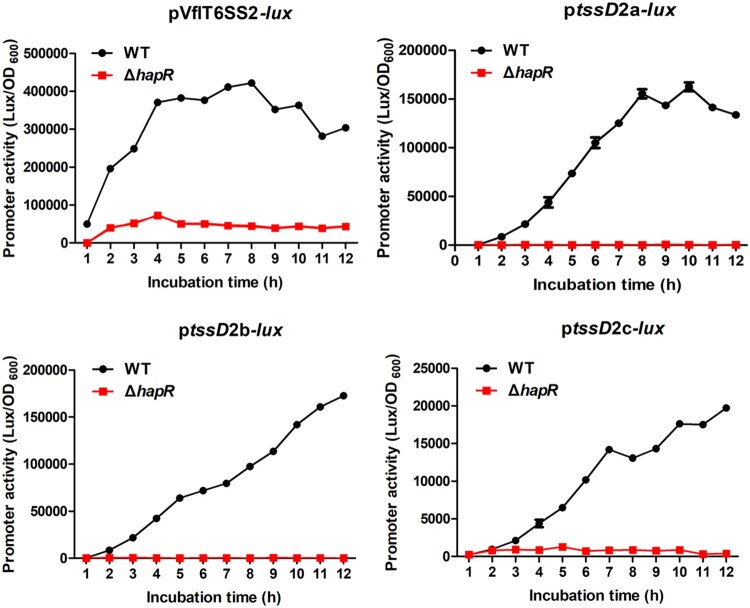
HapR modulates the promoter activities of VflT6SS2 clusters. Overnight cultures of *V. ﬂuvialis* 85003 WT and Δ*hapR* mutant containing pVflT6SS2*-lux*, p*tssD*2a*-lux*, p*tssD*2b*-lux*, or p*tssD*2c*-lux* were 1:100 diluted in fresh LB and incubated at 30°C with shaking. At the designated time points, the luminescent activities were measured and reported as light units/OD_600_.

To determine whether HapR regulates VflT6SS2 function at transcriptional level, we individually introduced pVﬂT6SS2-*lux,* p*tssD*2a-*lux,* p*tssD*2b-*lux*, and p*tssD*2c-*lux* reporter plasmids which respectively containing the promoter regions of VﬂT6SS2 major cluster and three orphan clusters [24] into WT or Δ*hapR* strain*,* and measured the heterogeneous promoter-driven luminescence activities. As shown in Figure 4, compared with the WT, all the promoter activities of pVﬂT6SS2-*lux*, p*tssD*2a-*lux*, p*tssD*2b-*lux*, and p*tssD*2c-*lux* in Δ*hapR* are very low, indicating that HapR induces the major and orphan cluster transcriptions to activate VflT6SS2 function. However, whether HapR directly works on the promoter regions of VflT6SS2 still needs to be determined.

### HapR physically interacts with the promoter region of the major cluster of VflT6SS2

**Figure 5.**
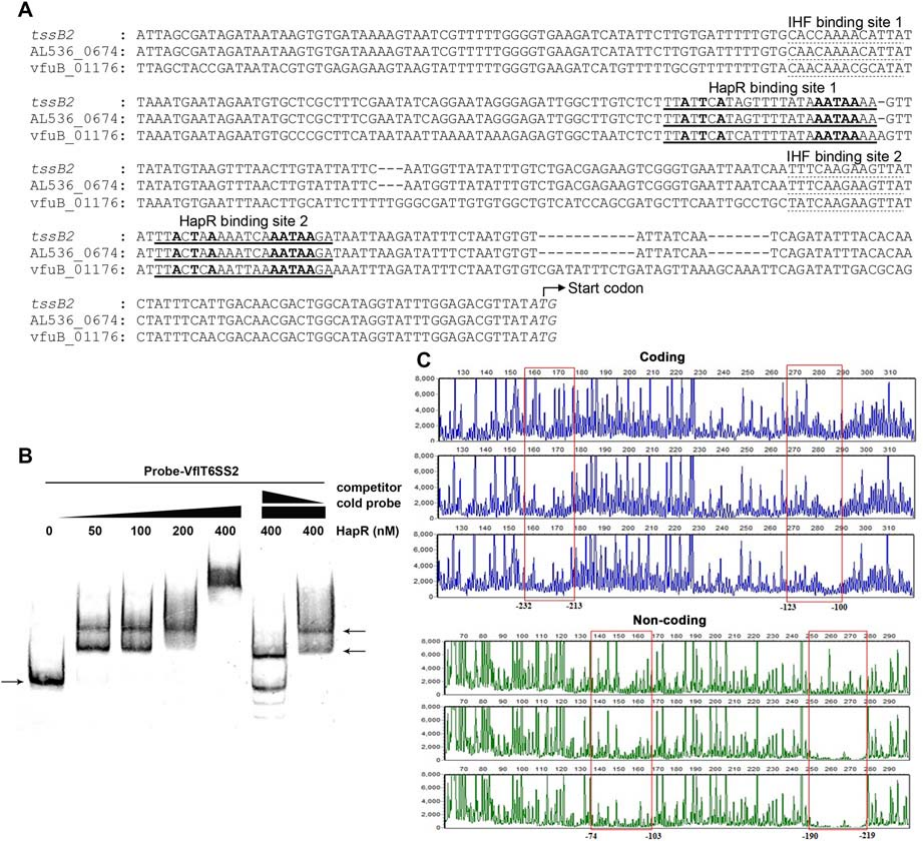
HapR physically binds to the promoter region of VflT6SS2 major cluster. (A) Nucleotide sequence analysis of the promoter region of VflT6SS2 major cluster. Nucleotide sequences of the proximal regions of *tssB*2 (*V. fluvialis* 85003), AL536_06745 (*V. fluvialis* 33809) and vfuB_01176 (*V. furnissii* NCTC11218) are aligned. The HapR binding sites are underlined and the conserved bases according to the Motif 1 binding sites [41] are shown in bold. Dotted underlined sequences show the IHF binding sites characterized previously [24]. Bases in italics show the beginnings of translation of *tssB*2, AL536_06745, and vfuB_01176. Dashes show gaps introduced to maximize alignment. (B) HapR binding to the promoter region of VflT6SS2 major cluster. EMSA was performed as described in the “Materials and Methods.” A biotin-labelled 450-bp DNA probe (20 ng) was incubated with increasing amounts of purified HapR protein. For competition analysis, the same but unlabelled probe was included at 10- or 50-fold concentrations relative to the labelled probes. The left arrow indicated the free probe, whereas the right arrows referred to HapR-bound probes. (C) DNase I footprinting assay of HapR binding to the promoter region of VflT6SS2 major cluster. As described in the “Materials and Methods,” increasing amounts of purified HapR protein was incubated with FAM/HEX-labelled fragments of *tssB*2 promoter region, and then the fragments were digested with the optimized RQ1 RNase-Free DNase I (Promega, United States). Finally, the digested fragments were analysed, and the protected regions were boxed and marked with positions. The negative numbers at the bottom indicate the nucleotide positions relative to the translation start site (+1) of *tssB*2, the first gene of VflT6SS2 major cluster.

To clarify if HapR directly binds to VflT6SS2 major cluster and orphan clusters, we firstly analysed their promoter sequences to search for potential *cis-*acting element(s). The sequence identity of HapR in *V. fluvialis* and *V. cholerae* has up to 78%, so we reasoned that the reported HapR-binding site in *V. cholerae* could be used as a reference [40,41]. Indeed, we found two potential HapR binding sites, 5′-TTATTCATAGTTTTATAAATAAAA-3′ and 5′-TTACTAAAAATCAAATAAGA-3′, in the promoter region of VflT6SS2 major cluster which have high similarities to the Motif 1 binding site in *V. cholerae* [41]. The two binding sites are located at nucleotides −224 to −201 and −111 to −92 relative to the *tssB*2 (*vipA*) start codon, downstream of the two IHF binding sites previously identified in the promoter region [24] (Figure 5(A)).

To prove the direct binding of HapR to the two sites, we performed EMSA and DNase I footprinting assays with purified HapR protein. As displayed in Figure 5(B), two shifted bands were observed with 50 nM of HapR protein in the reaction mixture, and only one band with slower migration was retained when HapR concentration rose to 200 nM. However, the shifted bands were not detected when HapR was deprived from the reaction mixture. Furthermore, inclusion of the same, but unlabelled DNA fragment (10 fold competing cold probe) led to the depletion of the retarded bands with the highest molecular weight, and the appearance of free probe band with more cold probe (50 fold).

Next, DNase I footprinting assay (Figure 5(C)) further confirmed the existence of two HapR binding sites at the promoter region of VflT6SS2 major cluster by displaying two protected regions against DNase I digestion, one from −232 to −219 and another from −123 to −74 (relative to the start codon). Both of the protected regions cover the predicted Motif 1binding sites.

### VflT6SS2 orphan clusters underwent HapR-involved dual regulation

Though VﬂT6SS2 harbours three orphan *tssD*2-*tssI*2 (*hcp*-*vgrG*) clusters, namely *tssD*2_a-*tssI*2_a, *tssD*2_b-*tssI*2_b, and *tssD*2_c-*tssI*2_c, we focused on the first two clusters in this study as they have the higher expression level in *V. fluvialis* (Figure 4) [23,24]. Since HapR transactivates the VﬂT6SS2 major cluster (Figure 4), the low expression of p*tssD*2a-*lux* and p*tssD*2b-*lux* reporter fusions in Δ*hapR* might be resulted from reduced expression of T6SS transcriptional regulator VasH, which resides in the VﬂT6SS2 major cluster operon and is absolutely required for the transactivation of three orphan clusters [24].

**Figure 6.**
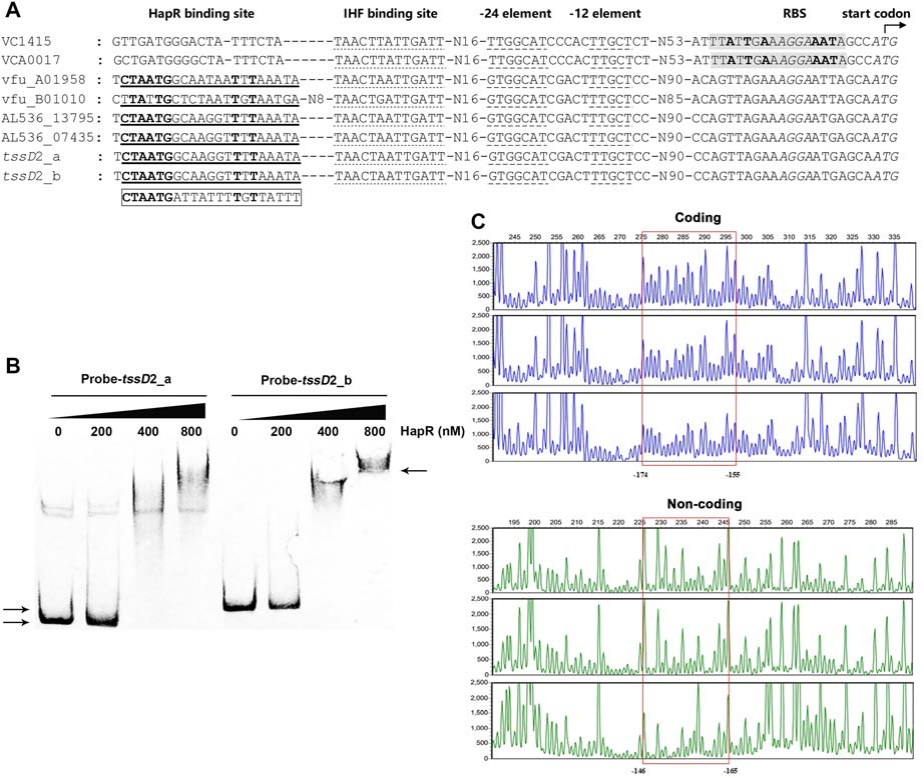
HapR directly binds to the promoter regions of VflT6SS2 orphan cluster. (A) Characteristics of the promoters of *hcp* homologs in different *Vibrio* species. The nucleotide sequences of *hcp* promoter regions in *V. cholerae* (VC1415, VCA0017), *V. furnissii* (vfuA_01958, vfuB_01010), *V. ﬂuvialis* 33809 (AL536_13795, AL536_07435), and 85003 (*tssD*2_a, *tssD*2_b) were compared. The ribosome binding site (RBS) and ATG start codon were shown in italics. Dashed underlined sequences show σ^54^ (−12/−24 element) consensus ones. Dotted underlined show IHF binding sites characterized previously [24]. The shaded nucleotides represent the Motif 1 HapR binding site identified in *hcp* promoters in *V. cholerae* [41]. The predicted Motif 2 binding sites were underlined. The boxed sequence at the left bottom shows the experimentally confirmed HapR binding site in *V. cholerae hapR* (VC0583) promoter [40]. (B) HapR binding to promoter regions of VflT6SS2 orphan cluster. EMSA was performed as described in the “Materials and Methods.” The biotin-labelled probe (20 ng) of *tssD*2_a or *tssD*2_b promoter regions was incubated with increasing amounts of purified HapR protein. The left arrows denoted the free probes, whereas the right arrow pointed to the HapR-bound probe. (C) DNase I footprinting assay of HapR binding to the promoter region of *tssD*2_a. As described in the “Materials and Methods,” increasing amounts of purified HapR protein was incubated with FAM/HEX-labelled fragments of *tssD*2_a promoter region, and then the fragments were digested with the optimized RQ1 RNase-Free DNase I (Promega, United States), and the resulting DNA fragments of different lengths were analysed. The protected regions were boxed and their binding positions were marked. The negative numbers indicate the nucleotide positions relative to the translation start site (+1) of *tssD*2_a gene.

To make clear if HapR is able to directly regulate the promoter activities of *tssD*2_a (*hcpA*) and *tssD*2_b (*hcpB*), we firstly examined the promoter sequences of *tssD*2_a (*hcpA*) and *tssD*2_b (*hcpB*) to look for possible HapR bind element. Interestingly, a potential Motif 2 binding site (5′-CTAATGGCAAGGTTTTAAATA-3′) but not Motif 1 binding site was identified in each of the promoter region [41], which positioned at nucleotides −180 to −160 relative to the *tssD*2_a (*hcpA*) and *tssD*2_b (*hcpB*) start codon, just upstream of the IHF binding site identified previously (Figure 6(A)) [24]. The Motif 1 and Motif 2 binding sites are quite different in terms of the conserved nucleotides and the binding sequence length [41]. The Motif 2 binding sites predicted in *V. fluvialis* have a high homology to that identified in *V. cholerae hapR* (VC0583) promoter by sharing 8 common bases at the highly conserved positions (Figure 6(A)) [40,41]. To determine if these are authentic HapR binding sites, we performed EMSA and DNase I footprinting assays with 5′ biotin-labelled or fluorescent FAM-labelled probe encompassing the predicted Motif 2 binding site. As displayed in Figure 6(B), retarded smears were observed with the addition of 400 nM or 800 nM HapR protein in the reaction mixture for both *tssD*2_a and *tssD*2_b probes, which are indicative of direct binding. DNase I footprinting assay revealed a HapR protected region which extends from −174 to −146 relative to the start codon and encompasses the predicted Motif 2 binding site (−180 to −160). It’s noteworthy that the Motif 2 binding site in the orphan cluster promoter displays lower affinity than the Motif 1 binding site in major cluster promoter as the former required a about 8-fold more HapR protein to obtain comparable retarded bands.

**Figure 7.**
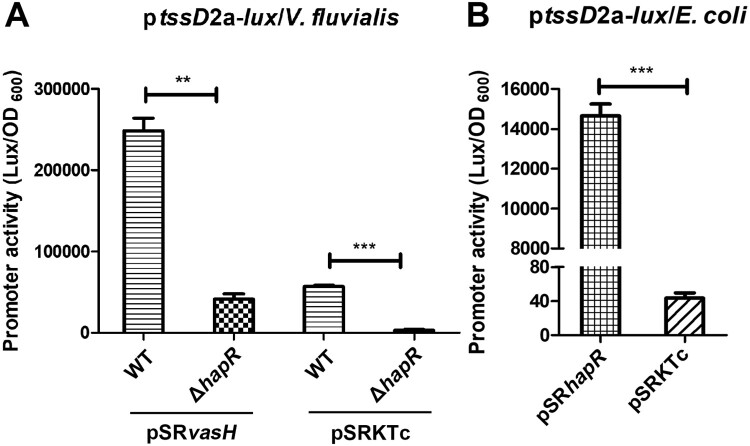
HapR directly regulates *tssD*2_a promoter activity independent of VasH. (A) The activity of p*tssD*2a-*lux* in *V. fluvialis* WT and Δ*hapR* mutant containing either pSR*vasH* or pSRKTc vector. Overnight cultures of the strains were1:100 diluted in fresh LB with IPTG induction at 30°C with shaking. The luminescent activity was measured and calculated as light units/OD_600_. Statistical significance was determined by unpaired two-tailed Student’s *t* test. **P = 0.0003; ***P < 0.0001. (B) The activity of p*tssD*2a-*lux* in *E. coli* SM10*λpir* with pSR*hapR* or pSRKTc. Overnight cultures of the above *E. coli* strains were 1:100 diluted in fresh LB and incubated at 30°C with shaking. The luminescence was measured and reported as light units/OD_600_. Statistical significance was detected using unpaired two-tailed Student’s *t* test. ***P < 0.0001.

To investigate the functional significance of direct HapR binding to VflT6SS2 promoter region, we introduced the IPTG-induced VasH over-expressed pSR*vasH* or its control empty vector [24] into WT or Δ*hapR* strain to eliminate the impact of possible differentially expressed endogenous *vasH*, and then the promoter activity of p*tssD*2a-*lux* fusion was measured. As shown in Figure 7(A), luminescence activity of p*tssD*2a-*lux* significantly reduced in Δ*hapR/*pSRKTc, however, the introduction of pSR*vasH* into Δ*hapR* raised the luminescence signal to a level comparable to that in WT*/*pSRKTc, but still greatly lower than in WT*/*pSR*vasH.* This result illustrates that high expression of exogenous VasH failed to recover the effect caused by HapR deletion, suggesting the existence of a direct effect of HapR on p*tssD*2a-*lux* expression. To double confirm our result, we measured the luminescence activity of p*tssD*2a-*lux* in *E. coli* strain SM10*λpir* which does not have T6SS and therefore is lack of VasH regulator. As expected, the luminescence activity of p*tssD*2a-*lux* was extremely low in SM10*λpir* containing control plasmid pSRKTc, but significantly high in SM10*λpir* with over-expressed pSR*hapR*, further supporting that HapR is capable of regulating VflT6SS2 orphan cluster activity independent of VasH expression.

## Discussion

T6SS is a newly discovered protein secretion system in Gram-negative bacteria, which is widely involved in bacterial pathogenicity and environmental survival through affecting biofilm formation, extracellular protease expression, motility, virulence, stress response, colonization competition, host–pathogen interaction and others [16,22,42–44]. T6SSs are also subjected to precise regulation which is intelligently integrated into the existing regulatory pathways and signal transduction devices, such as two-component system, quorum sensing system, ﬂagella system, nucleoside-scavenging pathway, chitin-induced competency pathway, and post-translational regulation [22,25,45]. In this study, we proved that the expression of VflT6SS2 is regulated by CqsA/LuxS-HapR QS circuit in *V. fluvialis*, an emerging foodborne pathogen which causes outbreaks and sporadic cases of acute diarrhoea. We showed that the Hcp expression and secretion were negatively regulated by QS response regulator LuxO but positively regulated by HapR, another major QS regulator (Figure 1). The repression effect of LuxO is more prominent at low cell density (Figure 2) under which condition LuxO is supposed to be activated by phosphorylation leading to repression of the expression of its downstream target LuxR or HapR [31,39,46]. Therefore, we supposed that the repressed Hcp expression by LuxO is also due to the reduced expression of HapR in *V. fluvialis*. This conjecture is supported by the constitutive high level of HapR as reflected by the luminescence activity of cosmid pBB1 carrying the *V. harveyi luxCDABE* operon in Δ*luxO* (Figure 3(A)) and confirmed by the lack of Hcp expression and secretion in double mutant Δ*luxO*Δ*hapR* (Figure 3(B)). We concluded that both LuxO and HapR significantly modulate VflT6SS2 function, but LuxO works through targeting HapR expression. Currently, we do not know how LuxO regulates the expression or activity of HapR. Since phosphorylated LuxO was reported to activate small RNAs [25,47], it deserves to explore if these RNAs participate in this process in *V. fluvialis*.

LuxU locates upstream of LuxO in the QS cascade and is a cytoplasmic phosphotransferase which converges the upstream signals and transfers phosphate group to LuxO at low cell density in *V. cholerae* [31,39,46]. As the composition of CqsA/LuxS-HapR QS circuit in *V. fluvialis* is very similar to *V. cholerae* [36], at the beginning, we expected a similar Hcp expression and secretion profile in Δ*luxO* and Δ*luxU* strains. Beyond our expectation, the profiles of Hcp expression and secretion, as well as HapR activity in Δ*luxU* were much different from in Δ*luxO*. These results imply that LuxO probably has an alternative function which is LuxU-independent. Yet whatever the situation is needs further investigation.

In this study, we totally identified four HapR binding sites in the major and orphan clusters of VflT6SS2 (except *tssD*2_c which we did not check in this study), and HapR induces more than 2-fold promoter activity in the major cluster than in any one of the orphan clusters, probably because of the following two reasons: first, there are two HapR binding sites in the major cluster promoter; second, HapR has a higher affinity to its binding site in the major cluster as shown in our EMSA results. Currently, we do not know if there exists HapR binding site (s) in *tssD*2_c, and what is the sequence characteristics and conservation, how is its affinity to HapR, and why its promoter activity is approximately 8–20 fold lower than other three ones in response to HapR regulation (Figure 4), these answers need to be disclosed in the future studies.

We noticed that the two HapR binding elements are situated downstream of each of the two IHF binding sites previously identified in the major cluster (Figure 5(A)) [24], while closely positioned upstream of an IHF binding site in the orphan cluster (Figure 6(A)). Therefore, HapR probably activates VflT6SS2 expression through co-regulating its major cluster and orphan clusters with IHF, and the co-regulation mechanism is not conserved in *V. cholerae*, though its T6SS genetic composition and organization are very similar with in *V. fluvialis* [23,24]. In *V. cholerae,* HapR was reported to transcriptionally activate two *hcp* alleles [25,26,48], but sequence inspection reveals no potential HapR binding site in the promoter region of *vipA* (VCA0107), the first coding gene of the major cluster operon. Another difference is that the HapR binding site in *V. cholerae hcp* promoter is the Motif 1 site and located 4 bp upstream of start codon [41]. Though a roughly similar element with the same conserved bases exits in *V. fluvialis hcp* promoter, it does not function as a binding site, as evidenced by the identical promoter activities of reporter fusions with and without this element (data not shown). Though the benefit of possible co-regulation of major cluster and orphan clusters in *V. fluvialis* is not clear, we reasoned that this joint regulation may assure the rapid and maximal activation of *hcp* orphan clusters to meet the needs for large amount of Hcp protein which serves as the structural unit of the long tube of T6SS and effector chaperones.

QS is implicated in T6SS regulation in many *Vibrio* spp., but with differential outcomes. Though there exists diversity in the detailed regulatory mechanism, QS coordinates T6SS activation by repressing the T6SS at low cell density but activates it at high cell density in both *V. cholerae* [25,47] and *V. fluvialis*. While in fish pathogen *V. anguillarum*, Hcp expression is repressed by QS regulator VanT, a HapR homologue [43]. In *V. alginolyticus* and *V. parahaemolyticus,* there are two T6SSs which are oppositely regulated by QS. In *V. alginolyticus*, the Hcp1 expression of T6SS1 was positively and negatively regulated by QS regulators LuxO and LuxR, respectively [49], while T6SS2 was promoted by LuxR [50]. In *V. parahaemolyticus*, the regulator OpaR (HapR homologue) represses T6SS1 but activates T6SS2 [51,52]. So, although these *Vibrios* spp. are all water-borne organisms and naturally exit in aquatic environment, the regulatory effects of QS on T6SSs vary greatly in different *Vibrios*. The reason for the difference is still unclear, we deduce that it may be related to differential host pathogenicity and corresponding living microenvironment of different *Vibrios*, and is the consequence of long-term adaptive evolution.

In conclusion, our current study demonstrated that the CqsA/LuxS-HapR QS circuit in *V. fluvialis* makes use of LuxO and HapR to coordinate VflT6SS2 activation by repressing the VflT6SS2 at low cell density and activating the VflT6SS2 at high cell density. These processes are carried out through LuxO modulating HapR and HapR transactivating VflT6SS2 major and orphan clusters. Besides, our results indicate the possible existence of an alternative QS signalling pathway in *V. fluvialis* which seems diverge LuxU and LuxO signal cascade. All in all, our study here revealed a detailed regulative mechanism of CqsA/LuxS-HapR QS circuit on VflT6SS2 in *V. fluvialis* which greatly enriched our understanding of the diversity and universality of the crosstalk between QS system and T6SS in microorganisms.

## Supplementary Material

Figure_S1.tif

supplementary_materials_TableS1_primers_clean_copy.docx
