# Supplementary figures and images for: CqsA/LuxS-HapR Quorum sensing circuit modulates type VI secretion system VﬂT6SS2 in Vibrio fluvialis
Source: Emerg Microbes Infect. 2021 Mar 30;10(1):589–601. doi: 10.1080/22221751.2021.1902244 (PMC8018390; doi:10.1080/22221751.2021.1902244)

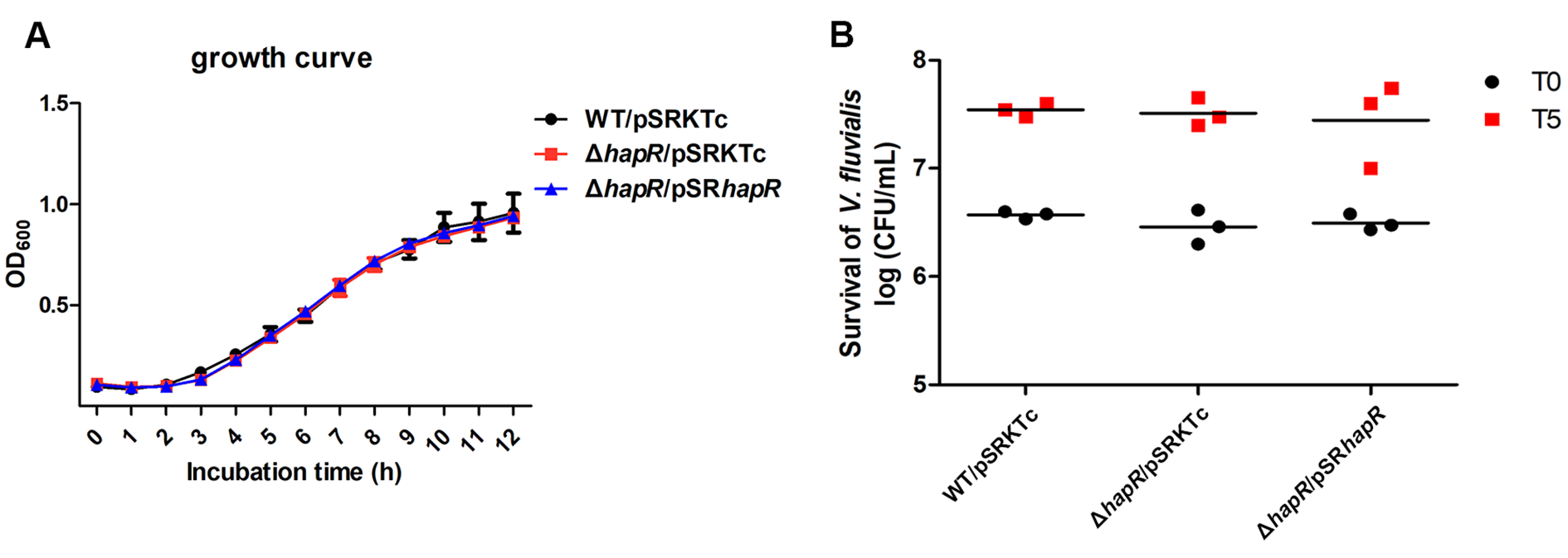

Supplement: Figure_S1.tif [file TEMI_A_1902244_SM1090.tif]
