# Supplementary material for: CqsA/LuxS-HapR Quorum sensing circuit modulates type VI secretion system VﬂT6SS2 in Vibrio fluvialis
Source: Emerg Microbes Infect. 2021 Mar 30;10(1):589–601. doi: 10.1080/22221751.2021.1902244 (PMC8018390; doi:10.1080/22221751.2021.1902244)
Supplement: supplementary_materials_TableS1_primers_clean_copy.docx [file TEMI_A_1902244_SM1089.docx]

Primers used in this study.

| Primers | sequence (5' to 3') |
| --- | --- |
| hapR-*Nde*I  hapR-*Xho*I  hapR-*Xho*I-1 | GGAATTCCATATGGACGCATCTATAGAG  CCGCTCGAGGTGATCGCGTTTATAGATG  CCGCTCGAGTTAGTGATCGCGTTTATAG |
| Vfl-cqsA-F1-up-*Xho*I  Vfl-cqsA-F1-dn  Vfl-cqsA-F2-up  Vfl-cqsA-F2-dn-*BamH* I  hcp-qPCR-F-com  hcp-qPCR-R-com  VF-recA-qPCR-up  VF-recA-qPCR-dn  HcpA-up-Biotin  HcpA-dn-Biotin  HcpB-up-Biotin  T6SS2-up-Biotin  T6SS2-dn-Biotin  vipA-M13F1-FAM  vipA-M13R-HEX  HcpA-M13F1-FAM  HcpA-M13R-HEX | GGCTCGAGTCACTGGCAATCGGTGGT  AACTGATGCCTAACCACGGGAGACCTAGCT  CCCGTGGTTAGGCATCAGTTTTCATGGTGA  CGGGATCCTTCATGTTACCGCTGGTG  TCGGCGATTCATTCGTT  CAGTTCAACCGTCGTCATCT  ACCGAGTCAACGACGATAAC  GATGAACTGCTGGTGTCTC  TGAGAATAGCCTTCCTTGAC  GAGTTTGACCTTCGATAGAG  GTGCCACCTTTGGCTACGTT  ACCATGATCTGTTCTGGGAT  TTAGGAGCTACACTTCCTTC  GTAAAACGACGGCCAGTGCGATAGATAATAAGTGTG  CAGGAAACAGCTATGACATGCGCTCTTTAGGAG  GTAAAACGACGGCCAGTCACCGCTGCTTGATCC  CAGGAAACAGCTATGACCCTGCAGTGATTAGAC |

Note: The restriction enzyme sites were underlined. Dashed-underlines indicate the FAM/HEX label.
